# The Polycomb group gene *rnf2* is essential for central and enteric neural system development in zebrafish

**DOI:** 10.3389/fnins.2022.960149

**Published:** 2022-09-01

**Authors:** Gang Feng, Yuhua Sun

**Affiliations:** ^1^Institute of Hydrobiology, Chinese Academy of Sciences, Wuhan, China; ^2^College of Advanced Agricultural Sciences, University of Chinese Academy of Sciences, Beijing, China

**Keywords:** PRC1, *rnf2*, ENS, CNS, neural crest, neural precursor cells

## Abstract

The development of central nervous system (CNS) and enteric nervous system (ENS) is under precise and strict control in vertebrates. Whether and how the Polycomb repressive complex 1 (PRC1) is involved in it remain unclear. To investigate the role of PRC1 in the nervous system development, using CRISPR/Cas9 technology, we have generated mutant zebrafish lines for the *rnf2* gene which encodes Ring1b, the enzymatic component of the PRC1 complex. We show that *rnf2* loss of function leads to abnormal migration and differentiation of neural crest and neural precursor cells. *rnf2* mutant embryos exhibit aganglionosis, in which the hindgut is devoid of neurons. In particular, the formation of 5-HT serotonin neurons and myelinating glial cells is defective. Furthermore, ectopic expression of ENS marker genes is observed in forebrain of *rnf2* mutant embryos. These findings suggest that the *rnf2* gene plays an important role in the migration and differentiation of neural precursor cells, and its absence leads to abnormal development of ENS and CNS in zebrafish.

## Introduction

During neural development, the migration and differentiation of progenitor cells must be precisely regulated to ensure that various neuronal cell types are generated in a tightly controlled manner. Enteric nervous system (ENS) is completely derived from neural crest cells (NCs), a transitional cell group with multiple differentiation potential (Nagy and Goldstein, 2017). The NCs can also differentiate into craniofacial tissues, pigment cells, neurons, and glial cells from sensory ganglion, and sympathetic ganglion (Rogers et al., 2012). In zebrafish, the ENS is derived from part of the vagal crest cells (Elworthy et al., 2005; Kenneth and Michael, 2005; Olden et al., 2008; Nagy and Goldstein, 2017; Julia, 2018). Neural crest stem cells (NCSCs) form between the non-neural ectoderm and the edge of the neural plate under the induction of signaling molecules (Avantaggiato et al., 1994). After the specification, they leave the neural crest and migrate, and a portion of them finally colonize the intestine. Once they reach the gut, these cells can be named as enteric NCSCs, also called the ENS progenitor cells, and are capable of differentiating into the neurons of the ENS (Jain et al., 2004). The ENS contains more than 1 million neurons, which can be divided into at least 18 functional subtypes, including four main neuron types: motor neurons, intrinsic primary afferent neurons, enteric neurons, and interneurons (Furness, 2000; Simon, 2001; Schemann, 2005).

Previous studies have shown that the migration and differentiation of NCSCs are under control of signal pathways and transcription factors, disruption of which may lead to abnormal development of ENS. For instance, in mice and birds, Sox10, Foxd3, Phox2b, Pax3, and other transcription factors have been shown to play key roles in the development of ENS (Jhiang et al., 1996; Hansford and Mulligan, 2000; Shepherd et al., 2004; Kwon et al., 2011). Patients with *PHOX2B* mutation show deficiency of ganglion cells (Benailly et al., 2010). In mice, *Ret* and *Gdnf* can regulate the survival, proliferation, and differentiation of ENS precursor cells (Shepherd et al., 2004; Amiel and Lyonnet, 2008). Either *Ret* or *Gdnf* gene knockout results in enteric aponeurosis ganglion cell and renal dysplasia (Wilson et al., 2002). In zebrafish, ablation of either *ret* or *gdnf* gene leads to the defective formation of enteric ganglion cells (Shimotake et al., 2001).

The central nervous system (CNS) of zebrafish originates from the ectodermal epithelium on the dorsal side of the embryo, called the neural plate (Wilson et al., 2002). During the development of zebrafish CNS, the primitive neural tube gradually develops into a mature system with more function-specific cell types (Kimmel et al., 1995; Wilson et al., 2002). The cells in the anterior region of the neural tube proliferate and differentiate into the primordia of the fore-, mid-, and hindbrain, while the posterior neural tube differentiates into the spinal cord (Wilson et al., 2002; Schier and Talbot, 2005; Durston and Zhu, 2015).

The Polycomb group (PcG) is composed of a variety of transcription inhibitors, mainly through epigenetic regulation of its target genes at the chromatin level (Bulyzhenkov et al., 1974; Croce and Helin, 2013). The PcG proteins primarily form two principal complexes, the Polycomb repressive complex 1 (PRC1), and the Polycomb-repressive complex 2 (PRC2). PRC2 is responsible for the trimethylation of Lys27 on histone H3 (H3K27me3) *via* the enzymatic subunit EZH1 or EZH2, while PRC1 catalyzes the ubiquitination of Lys119 on histone H2A (H2AK119ub) through the E3 ligase RING1. The biological functions of PRCs are under intensive investigation and are believed to be involved in stem cells maintenance, cell differentiation, cell cycle, aging, × chromosome inactivation, and tumorigenesis (Gil and O’Loghlen, 2014; Dupret et al., 2016; Almeida et al., 2017; Schuettengruber et al., 2017; Zhao et al., 2017; Chan et al., 2018; King et al., 2018). Two homologous subtype genes *Ring1a* and *Ring1b* have been found in mammalian genome (Schoorlemmer et al., 1997; Vidal, 2009). Knockout of *Ring1b* leads to the cessation of gastrulation and death of mouse embryos (Voncken et al., 2003; Napoles et al., 2004). *Ring1a* and *Ring1b* in mouse embryonic stem cells (ESCs) maintain its undifferentiated state by inhibiting differentiation genes (Endoh et al., 2008; van der Stoop et al., 2008). Patients with RING1 dysfunction show neurogenic psychosis, developmental abnormalities, and cognitive impairment (Pierce et al., 2018), suggesting that it has an important function in neural development. Zebrafish has only one *rnf2* gene, which is high homology to human *RING1B* (Le Faou et al., 2011). *rnf2* mutant zebrafish embryos show pleiotropic phenotypes, including lack of pectoral fin, craniofacial cartilage defects, edema, and stringy heart with abnormal sarcomere assembly (Velden et al., 2012, 2013; Chrispijn et al., 2019; Peng et al., 2021). However, the role of *rnf2* in neural development remains unclear.

In this work, we hypothesize that the *rnf2* gene of PRC1 plays key role in the development of central and ENS s. By generating *rnf2* mutant zebrafish, we show that *rnf2* loss of function affects the migration and differentiation of neural precursor cells during the development of both ENS and CNS. Our results provide important insights into the roles of *rnf2* in embryonic development and diseases.

## Materials and methods

### Zebrafish strain and husbandry

The AB strain zebrafish and their embryos were maintained and raised in recirculation system at 28.5°C under a 14-h light, 10-h dark photoperiod. Developmental stages of zebrafish embryos were determined as previously described (Kimmel et al., 1995).

### Generation of the *rnf2* mutant line

The zebrafish *rnf2* mutants were generated using the CRISPR/Cas9 system (Hwang et al., 2013; Jao et al., 2013; Peng et al., 2021). The guide RNA (gRNA) was designed targeting the exon 3 of the *rnf2* gene (Peng et al., 2021). Embryos at the 1-cell stage were co-injected with 200 ng/μL Cas9 mRNA and 80 ng/μL gRNA. The genomic DNA of 30 embryos at 24 hpf was extracted and subjected to PCR amplification. The DNA fragment containing the *rnf2* target site was amplified by PCR using the primers 5′-TTGAGGTAGTTGCTCCCAAAG-3′ and 5′-GGCATTCCTTGGTGGTCATA-3′, and the genotype was determined by DNA sequencing.

### Whole-mount *in situ* hybridization

Embryos at different developmental stages were sampled and fixed in 4% paraformaldehyde (PFA) at 4°C overnight and then transferred in 100% MeOH. Whole-mount *in situ* hybridization was carried out according to a standard protocol (Thisse and Thisse, 2008). The DIG-labeled anti-sense probes were generated using a DIG RNA Labeling Kit (SP6/T7) (Roche). INT/BCIP (Roche) were used as alkaline phosphatase substrates. The primers of the probes were showed in the table of Supplementary Table S1. For embryos at or after 48 hpf, the homozygotes were separated from their siblings according to their heart edema and pectoral fin phenotype. For embryos before 48 hpf, as it was difficult to separate homozygous mutants from the heterozygous and wild-type siblings, WISH was performed for all progeny of *rnf2*^±^ parents. After the WISH, each embryo was photographed and genotyped separately. The photographs were taken under a stereomicroscope (Leica Z16 APO) with a digital camera (Leica DFC450). The number and phenotype of embryos in each group were recorded, and then the offspring produced by *rnf2*^±^ self-cross were genotyped.

### Generation of *phox2b*: Enhanced green fluorescent protein transgenic line

We cloned a *phox2b*-promoter into pT2AL200R150G according to the Tol2 methods (Asakawa and Kawakami, 2009). The promoter contains approximately 4.8 kb of sequence upstream of transcription start site in the *phox2b gene*.^1^ Next, we co-injected linearized plasmid DNA and transposase mRNAs into 1-cell stage zebrafish embryos, and raised the injected embryos to adulthood. The injected embryos were crossed with wild type fish, and day 1 F_1_ embryos were screened for GFP under a dissecting microscope MZ 16FA (Leica). Embryos with GFP expression were raised up to adulthood. F_1_ adults were crossed to obtain F_2_ embryos with stable expression of GFP.

### Whole-mount immunostaining

For immunohistochemistry processing, embryos were fixed overnight at 4°C in 4% paraformaldehyde (PFA). Next day, embryos were washed three times in 1 × PBS and then transferred into tubes with 100% MeOH. Fluorescent immunostaining for 96 hpf embryos was performed using the polyclonal zebrafish Rnf2 antiserum (1:300). The DyLight 488 goat anti-rabbit IgG (1:350, Abbkine, United States) was used as secondary antibody. The neurons were detected by using rabbit anti-HuC/D (1:200) as primary antibody and DyLight 555 goat anti-mouse IgG (1:350, Abbkine, United States) as secondary antibody. For whole-mount immunostaining, embryos were digested in PBS containing 10 g/ml proteinase K, 0.1% Tween20 and blocked in PBS containing 10% normal goat serum, 0.5% DMSO and 0.3% Triton X-100 (Wang et al., 2013). Images were taken with a confocal laser scanning microscope (Leica SP8 DLS).

### Image, quantification, and statistical analysis

Images of fluorescent signals were taken with a confocal laser scanning microscope (Leica SP8 DLS). The signal density of the images was analyzed by Image J software (National Institutes of Health). The values are presented as mean ± SEM. The *p*-values were calculated by Origin 9.0 with two-tailed Student’s test, * represent *p* ≤ 0.05.

## Results

### *rnf2* is expressed in enteric nervous system and central nervous system of zebrafish embryos

**FIGURE 1:**
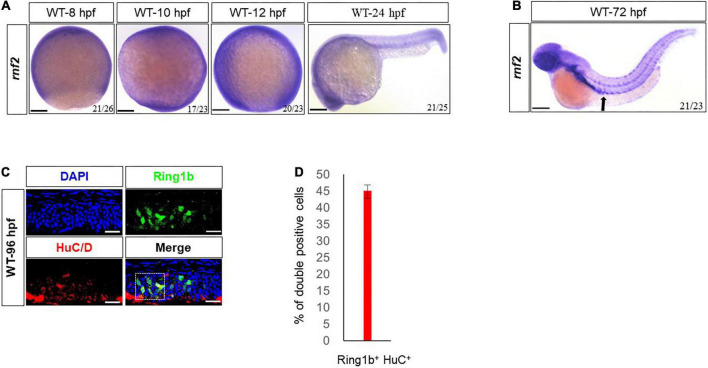
Expression patterns of *rnf2* in zebrafish embryos. **(A)** Whole mount *in situ* hybridization (WISH) of *rnf2* at the indicated time points. **(B)** The expression of *rnf2* at 72 hpf WT embryos. Black arrows showing the expression of *rnf2* in the gut of zebrafish embryos. **(C)** Immunohistochemistry images of *Rnf2* and HuC/D in zebrafish gut (40 × oil, embryos direction: anterior is to the left). The overlapping of Rnf2 and HuC/D expression is marked by the white box. **(D)** Quantitation of percentage of Rnf2/HuC double positive cells (*n* = 29). The experiments were repeated at least three times. Scale bar: 0.1 mm.

The expression pattern of *rnf2* during early embryogenesis has been described (Figure 1A; Velden et al., 2012). In addition to the brain, we found that *rnf2* was abundantly expressed in gut regions at 72 hpf (Figure 1B), suggesting that it may be involved in the development of ENS.

To confirm this, double fluorescence *in situ* hybridization for Rnf2 and the pan neural marker HuC/D was performed in 96 hpf embryos. The results showed that Rnf2 was expressed in the gut and its surrounding environment, and was colocalized with a portion of HuC/D^+^ neuronal cells (Figures 1C,D). These data suggested that *rnf2* was involved in the development of ENS and CNS in zebrafish embryos.

### *rnf2* is required for the migration of enteric neural precursor cells

To investigate the role of the *rnf2* gene, we generated *rnf2* mutant zebrafish using the CRISPR/Cas9 technology (Peng et al., 2021). The 5 bp deletion mutant line was used for the most of the experiments. No obvious phenotypes between WT (Wild type) and mutant embryos were observed at 24 hpf (Peng et al., 2021). After 72 hpf, *rnf2* mutants displayed pleiotropic phenotypes, including craniofacial defects, cardiac edema, and lack of pectoral fins (Velden et al., 2012, 2013; Chrispijn et al., 2019; Peng et al., 2021). No Ring1b protein was detected in *rnf2*^–/–^ embryos, and as expected, H2AK119ub level was markedly decreased. The *rnf2*^–/–^ embryos usually die within a week (Peng et al., 2021).

**FIGURE 2:**
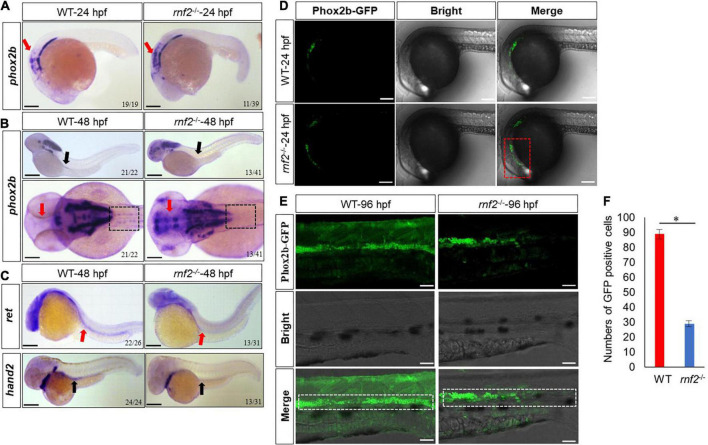
The effect of *rnf2* deficiency on the migration of enteric neural precursor cells in zebrafish embryos. **(A)** The expression of *phox2b* in *rnf2*^–/–^ and WT embryos at 24 hpf. Red arrows showing the reduced expression of *phox2b* in intestinal bulb of mutant embryos. Scale bar = 0.1 mm. **(B)** The expression of *phox2b* in *rnf2*^–/–^ and WT embryos at 48 hpf. Black arrows showing the reduced expression of *phox2b* in the gut of mutant embryos, red arrows showing the ectopic expression of phox2b in the brain regions. The dashed black box showing the compromised migration of ENPCs. Upper: lateral view; bottom: dorsal view. **(C)** The expression of *ret* and *hand2* in *rnf2*^–/–^ and WT embryos at 48 hpf. Black arrows showing the reduced expression of *hand2* in the gut of mutant embryos; red arrows showing the reduced expression of *ret*. The dashed black box showing the compromised migration of ENPCs. Upper: lateral view; bottom: lateral view. **(D)** The expression of Phox2b-GFP in the brain regions of *rnf2*^–/–^ and WT embryos at 24 hpf (40 × oil, lateral view). The numbers of samples were 28 and 24 in 24 hpf WT and *rnf2*^–/–^ embryos, respectively. The red dashed box showing the expansion of GFP signals. **(E)** The expression of Phox2b-GFP in gut regions of *rnf2*^–/–^ and WT embryos at 96 hpf (40 × oil, lateral view and head is to the left). The white dashed box showing the different distribution of GFP^+^ signals in the gut of WT and *rnf2*^–/–^ embryos at 96 hpf. The numbers of samples were 30 and 27 in 96 hpf WT and rnf2^–/–^ embryos, respectively. **(F)** Quantitation of GFP positive cells in **(E)**. The star indicates significant differences at *p* ≤ 0.05. The experiments were repeated at least three times. Scale bar: 0.1 mm.

First, we investigated the role of *rnf2* in ENS development. During zebrafish ENS development, enteric neural precursor cells (ENPCs) migrate as two chains from the postotic vagal regions to the caudal end along the two sides of the gut (Olden et al., 2008). *Phox2b*, which is mainly expressed in neural precursor cells (Garcia-Barceló et al., 2003; Fitze et al., 2010; Kwon et al., 2011), can be used to mark the enteric neural precursors (Elworthy et al., 2005). In order to investigate whether *rnf2* is required for the migration of neural precursor cells, whole mount *in situ* hybridization (WISH) was performed for *phox2b*. At 24 hpf, *phox2b* mRNAs were detected in the hindbrain and vagal regions, and the expression was comparable between WT and mutant embryos (Figure 2A). In 48 hpf WT, *phox2b* was expressed in the vagal region and the anterior part of the gut (Figure 2B). In 48 hpf *rnf2* mutant, however, *phox2b* was mainly distributed in the vagal region, and barely detected in the gut, suggesting that the migration of Phox2b-positive ENPCs was defective (Figure 2B). Of note, *phox2b* was barely expressed in forebrain and eye regions in WT embryos, while it was ectopically expressed in mutant embryos.

Next, we performed WISH for more enteric neural precursor markers, such as *ret* and *hand2* (Figure 2C). The results showed that the expression of *ret* and *hand2 was* decreased significantly in the gut regions of 48 hpf mutant embryos, compared to the controls.

To further investigate this, we examined enteric neural crest migration by using the Tg [*phox2b*: enhanced green fluorescent protein (EGFP)] transgenic line. In 24 hpf WT transgenic embryos, the Phox2b-GFP^+^ cells were mainly confined to the position of intestinal bulb (Figure 2D). In 24 hpf mutant transgenic embryos, Phox2b-GFP^+^ cells were more broadly distributed. Then we examined embryos at 96 hpf, when the ENPCs had finished the migration along the gut (Olden et al., 2008). In WT embryos, the Phox2b-GFP^+^ cells were distributed throughout the whole gut. In mutant embryos, however, the Phox2b-GFP^+^ cells were detected only in the foregut and midgut but not in hindgut (Figure 2E). Consistently, the number of Phox2b-GFP positive cells was much smaller in hindgut of mutant embryos compared to controls (Figure 2F). These results indicated that loss of *rnf2* leads to abnormal migration of enteric neural precursors.

### *rnf2* loss of function decreases enteric neurons

**FIGURE 3:**
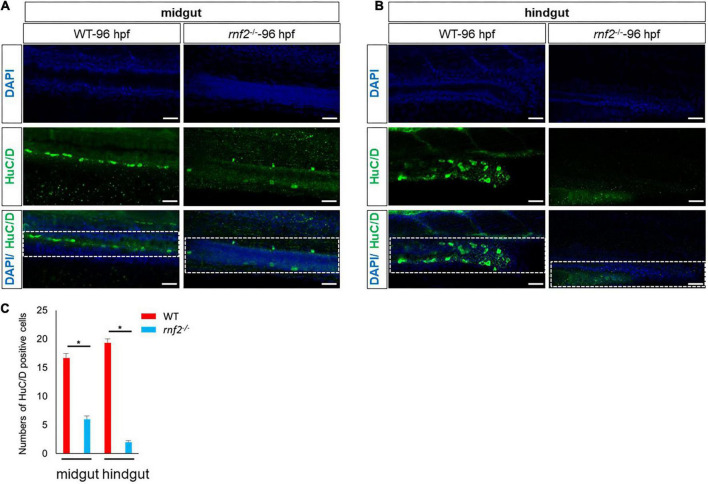
The deficiency of *rnf2* decreased the enteric neurons in zebrafish embryos. **(A)** Confocal images showing HuC/D positive enteric neurons in midgut of *rnf2*^–/–^ and WT embryos at 96 hpf (40 × oil, lateral view and head is to the left). The white dashed box showing the different distribution of HuC/D^+^ signals in the midgut of WT and *rnf2*^–/–^ embryos at 96 hpf. **(B)** Confocal images showing HuC/D positive enteric neurons in hindgut of *rnf2*^–/–^ and WT embryos at 96 hpf (40 × oil, lateral view and head is to the left). The white dashed box showing the different distribution of HuC/D^+^ signals in the hindgut of WT and *rnf2*^–/–^ embryos at 96 hpf. Scale bar: 0.1 mm. **(C)** Quantitation of HuC positive cells in **(A,B)**. The numbers of samples were 26 and 21 in 96 hpf WT and *rnf2*^–/–^ embryos, respectively. The star indicates significant differences at *p* ≤ 0.05. The experiments were repeated at least three times. Scale bar: 0.1 mm.

During the migration toward the gut, the ENPCs will gradually differentiate into different type of neurons (Oppenheim, 1991; Chelyshev et al., 2000). In zebrafish, the first differentiating enteric neurons are detected along the anterior gut at ∼55 hpf (Velden et al., 2012; Chrispijn et al., 2019), and by 74 hpf, HuC/D^+^ enteric neurons appear at the most caudal end of the gut, near the anus (Velden et al., 2012; Bergeron et al., 2013). Based on the above results, we speculated that the enteric neuron formation may be affected in the absence of *rnf2*. To test this, we performed whole mount immunohistochemistry for WT and *rnf2*^–/–^ embryos at 96 hpf, using antibodies against the pan-neuronal marker HuC/D. In WT embryos, HuC/D^+^ neurons were observed along the entire length of the gut, all the way to the anus (Figure 3). In *rnf2*^–/–^ embryos, however, HuC/D^+^ neurons were found only within the foregut and midgut regions (Figures 3A,B), indicating that loss of *rnf2* resulted in aganglionosis (Bergeron et al., 2013). Even in the midgut, the number of HuC/D^+^ neurons were smaller in *rnf2*^–/–^ embryos than in the controls (Figure 3C). These data suggested that *rnf2* is required for efficient colonization of ENPCs to the entire gut, and its loss causes aganglionosis.

### Loss of *rnf2* affects the specification of neural crest cells

**FIGURE 4:**
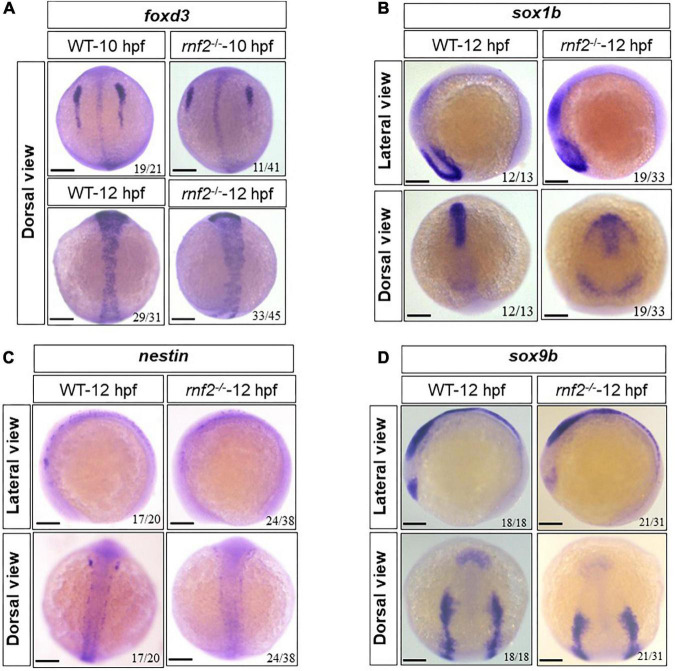
The effect of loss of *rnf2* on the specification of neural crest cells. **(A)** The expression of *foxd3* in *rnf2*^–/–^ and WT embryos at 10 and 12 hpf. Dorsal view. **(B)** The expression of *sox1b* in *rnf2*^–/–^ and WT embryos at 12 hpf. Upper: lateral view, bottom: dorsal view. **(C)** The expression of *nestin* in *rnf2*^–/–^ and WT embryos at 12 hpf. Upper: lateral view, bottom: dorsal view. **(D)** The expression of *sox9b* in *rnf2*^–/–^ and WT embryos at 12 hpf. Upper: lateral view, bottom: dorsal view. The experiments were repeated at least three times. Scale bar = 0.1 mm.

Next, we asked whether loss of *rnf2* affects the initiation and specification of NCs, by examining the expression of *foxd3*, *nestin*, *sox9b*, and *sox1b* in 10 and 12 hpf embryos (Li et al., 2002; Mahler and Driever, 2007; Hochgreb-Hägele and Bronner, 2013; Andrzejczuk et al., 2018). The results showed that at 10 hpf and 12 hpf, the expression of *foxd3*, *nestin*, *sox9b*, and *sox1b* was changed to different extent (Figure 4). In 10 hpf WT embryos, the *foxd3*-labeled NCs migrated linearly from the animal pole to the plant pole, and formed three distinct migration pathways. In *rnf2*^–/–^ embryos, there was a global similar expression of *foxd3*, but the migration chains of NCs were shorter, compared to controls (Figure 4A). At 12 hpf, the expression of *nestin*, *sox9b* was decreased significantly (Figures 4C,D), while *sox1b* showed an ectopic expression (Figure 4B). These observations suggested that the migration and distribution of NCs were disturbed in the absence of Ring1b.

**FIGURE 5:**
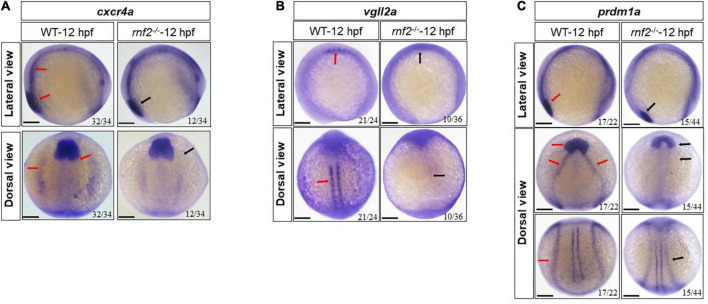
The effect of *rnf2* deficiency on differentiation of neural crest in zebrafish embryos. **(A)** The expression of *cxcr4a* in *rnf2*^–/–^ and WT embryos at 12 hpf. Upper: lateral view, bottom: dorsal view. **(B)** The expression of *vgll2a* in *rnf2*^–/–^ and WT embryos at 12 hpf. Upper: lateral view, bottom: dorsal view. **(C)** The expression of *prdm1a* in *rnf2*^–/–^ and WT embryos at 12 hpf. Upper: lateral view; middle: dorsal anterior view; bottom: dorsal posterior view. Red arrows showing the expression of *cxcr4a*, *vgll2a*, and *prdm1a* in 12 hpf WT embryos, black arrows showing the reduced expression of *cxcr4a*, *vgll2a*, and *prdm1a* in 12 hpf *rnf2*^–/–^ mutant embryos. The experiments were repeated at least three times. Scale bar = 0.1 mm.

Then we investigated the genes related to the early differentiation of NCs, including *prdm1a*, *cxcr4a*, and *vgll2a* (Olesnicky et al., 2010; Johnson et al., 2011; Chen et al., 2018). The expression of *prdm1a*, *cxcr4a*, and *vgll2a* was decreased significantly with varying degrees (Figure 5). In 12 hpf embryos, *cxcr4a* expression levels in neural crest progenitors were reduced in *rnf2*^–/–^ embryos (Figure 5A). *vgll2a* was strongly expressed along the two lines of the neural crest in WT embryos. In *rnf2*^–/–^ embryos, however, the expression of *vgll2a* was barely detected (Figure 5B). In 12 hpf WT embryos, *prdm1a* formed two expression chains around the vagal region, and exhibited 4 parallel chains around the neural crest and neural plate (Figure 5C). In 12 hpf *rnf2*^–/–^ embryos, the anterior expression of *prdm1a* in neural plate was hardly detected, and its posterior expression domains were closer to the midline, compared to the controls (Figure 5C). All these results indicated that *rnf2* knockout leads to abnormal migration and differentiation of NCs.

### *rnf2* loss of function affects central nervous system development

**FIGURE 6:**
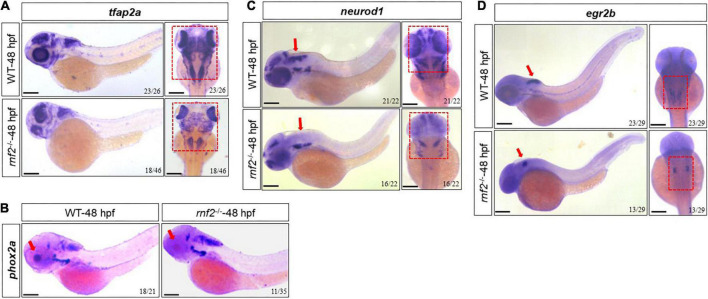
The effect of *rnf2* deficiency on central nervous system development in zebrafish embryos. **(A)** The expression of *tfap2a* in *rnf2*^–/–^ and WT embryos at 48 hpf. Left: lateral view; right: dorsal view of the brain. The red dashed boxes showing the different expansion of *tfap2a* in the brain regions of *rnf2*^–/–^ embryos at 48 hpf. **(B)** The expression of *phox2a* in *rnf2*^–/–^ and WT embryos at 48 hpf. The red boxes and arrows showing the loss of *phox2a* expression in the brain regions of mutant embryos. Lateral view. **(C)** The expression of *neurod1* in *rnf2*^–/–^ and WT embryos at 48 hpf. The red boxes and arrows showing the loss of *neurod1* expression in MHB and hindbrain of mutant embryos. Left: lateral view; right: dorsal view of the brain. **(D)** The expression of *egr2b* in *rnf2*^–/–^ and WT embryos at 48 hpf. The red arrows showing the reduced *egr2b* expression in MHB of mutant embryos. Left: lateral view; right: dorsal view of the brain. The experiments were repeated at least three times. Scale bar = 0.1 mm.

We next investigated whether *rnf2* loss of function disrupted normal CNS development in zebrafish embryos. To this end, we performed *in situ* hybridization against *neurod1*, *egr2b*, *tfap2a*, and *phox2a* in 48 hpf control and *rnf2* mutant embryos (Figure 6). *neurod1*, *egr2b*, *tfap2a*, and *phox2a* have been shown to be related to CNS development (Tahayato et al., 2003; Uehara et al., 2007; Zhang et al., 2017; Kousa et al., 2019; Song et al., 2020; Elliott et al., 2021). The results showed that the expression of *tfap2a* was slightly reduced in *rnf2*^–/–^ embryos compared to controls, especially in the middle and hindbrain regions (Figure 6A). *phox2a* was down-regulated in the eyes in the *rnf2*^–/–^ embryos compared to the controls (Figure 6B). *Tfap2a* and *Phox2a* are associated with the differentiation of adrenergic neurons and noradrenergic neurons, respectively (Kousa et al., 2019; Song et al., 2020). In WT embryos, *neurod1* was abundantly expressed in the forebrain, the midbrain, the hindbrain, and the midbrain-hindbrain boundary (MHB). In *rnf2*^–/–^ embryos, however, the expression domains of *neurod1* were lost in midbrain and MHB (Figure 6C). Consistently, in *rnf2*^–/–^ embryos, there was a reduced expression of *egr2b*, a marker of MHB (Figure 6D). These results suggested that *rnf2* is involved in the differentiation of neurons in the CNS.

**FIGURE 7:**
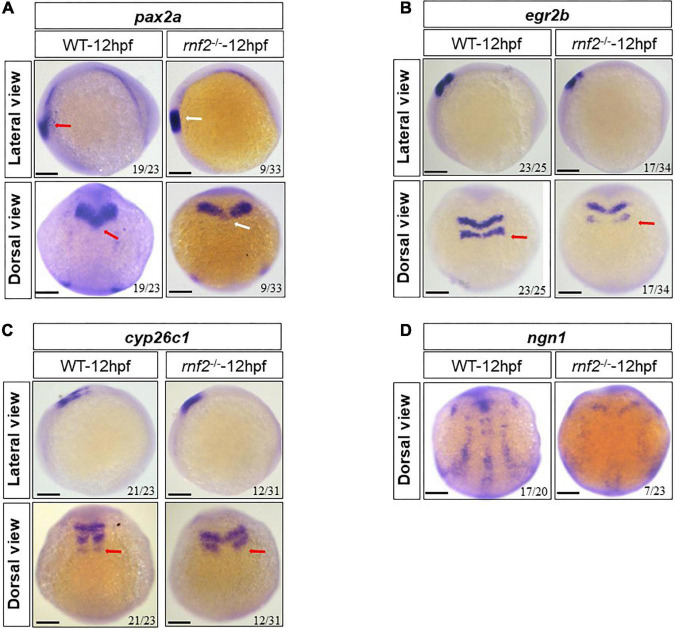
The effect of *ring1b* deficiency on the early development of brain in zebrafish embryos. **(A)** The expression of *pax2a* in *rnf2*^–/–^ and WT embryos at 12 hpf. Upper: lateral view, bottom: dorsal view. The red and white arrows, respectively, showing the different expression of *pax2a* in WT and *rnf2*^–/–^ embryos at 12 hpf. **(B)** The expression of *egr2b* in *rnf2*^–/–^ and WT embryos at 12 hpf. Upper: lateral view, bottom: dorsal view. The red arrows showing the different expression of *egr2b* in WT and *rnf2*^–/–^ embryos at 12 hpf. **(C)** The expression of *cyp26c1* in *rnf2*^–/–^ and WT embryos at 12 hpf. Upper: lateral view, bottom: dorsal view. The red arrows showing the different expression of *cyp26c1* in WT and *rnf2*^–/–^ embryos at 12 hpf. **(D)** The expression of *ngn1* in *rnf2*^–/–^ and WT embryos at 12 hpf. The experiments were repeated at least three times. Scale bar = 0.1 mm.

Next, we examined the expression of genes related to CNS progenitor development, including *pax2a*, *egr2b*, cyp26c1, and *ngn1* (Korzh et al., 1998; Tahayato et al., 2003; Uehara et al., 2007; Ota et al., 2016; Zhang et al., 2017). As shown in Figure 7, globally, *rnf2* knockout resulted in decreased expression of these marker genes. In WT embryos, *pax2a* displayed a V-type expression pattern. In *rnf2*^–/–^ embryos, the two expression domains along the midline were disconnected (Figure 7A). The expression of *egr2b* was decreased in 12 hpf *rnf2*^–/–^ embryos, especially in the hindbrain (Figure 7B). Similar results were observed for *cyp26c1* (Figure 7C). The expression of *ngn1* was markedly reduced in *rnf2*^–/–^ embryos compared to controls (Figure 7D).

**FIGURE 8:**
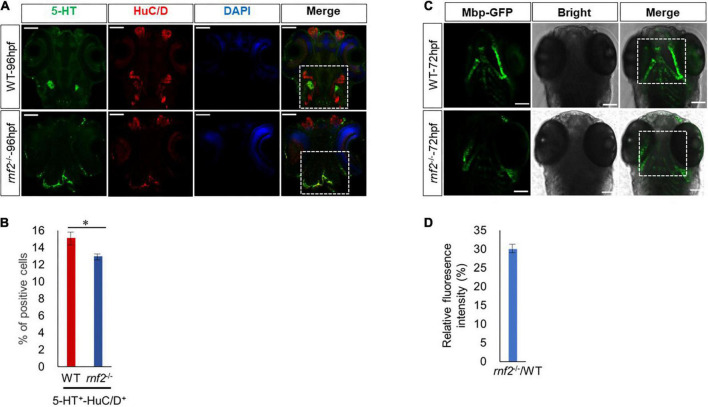
Loss of Rnf2 leads to neuronal differentiation defects in zebrafish embryos. **(A)** Confocal images showing the expression of HuC/D and 5-HT in zebrafish brain at 96 hpf (40 × oil). Dorsal view. The white dashed boxes showing the overlapping of HuC/D^+^ and 5-HT^+^ signals in the brain of WT and *rnf2*^–/–^ embryos at 96 hpf. **(B)** Quantitation of percentage of 5-HT/HuC double positive cells in **(A)**. The numbers of samples were 20 and 18 in 96 hpf WT and *rnf2*^–/–^ embryos, respectively. The star indicates significant differences at *p* ≤ 0.05. **(C)** Confocal images showing the expression of Mbp-GFP in zebrafish brain at 72 hpf (40 × oil). Dorsal view. The white dashed boxes showing the distribution of Mbp^+^ signals in the brain of WT and *rnf2*^–/–^ embryos at 72 hpf. **(D)** Quantitation of relative fluorescence intensity in **(C)**. The numbers of samples were 28 and 20 in 72 hpf WT and *rnf2*^–/–^ embryos, respectively. The experiments were repeated three times. Scale bar = 0.1 mm.

To investigate whether *rnf2* loss of function affects further differentiation of function-specific neurons, we analyzed 96 hpf WT and *rnf2*^–/–^ embryos by performing whole-mount immunofluorescence staining with antibodies against HuC/D and 5-HT (Figure 8A). 5-HT marks the serotonin neurons. In both WT and mutant embryos, 5-HT^+^ neurons were overlapped with a portion of HuC/D neurons. The expression of HuC/D was markedly reduced in the brain of *rnf2* mutants compared to the controls, particularly in the midbrain and hindbrain. In the brain of control embryos, 5-HT^+^ serotonin neurons were bilaterally localized and centralized distributed. By contrast, in *rnf2*^–/–^ embryos, 5-HT^+^ neurons were mis-localized and displayed a dispersed manner (Figure 8A). Furthermore, the percentage of 5-HT/HuC double positive cells was slightly smaller in *rnf2* mutant embryos than in controls (Figure 8B). These results indicated that 5-HT serotonin neuronal differentiation may still occur, but its migration was abnormal.

Finally, we asked whether loss of *rnf2* leads to defective formation of myelinating glial cells. To this end, we utilized a transgenic zebrafish line Tg (*mbp*: EGFP), in which the myelinating glial cells are labeled by GFP (von Jonquieres et al., 2013). In 72 hpf WT embryos, Mbp-GFP signals appeared in hindbrain and bilateral symmetry (Figure 8C). In 72 hpf *rnf2*^–/–^ embryos, however, Mbp-GFP signals in these regions were reduced and mis-localized (Figures 8C,D). These results showed that *rnf2* loss of function may affect the migration and differentiation of myelinating glial cells.

## Discussion

Polycomb group proteins (PcGs) are important epigenetic repressors that play important roles in the regulation of ESCs and hematopoietic stem cells (HSCs), cell fate determination, cell lineage restriction and organogenesis (Chittock et al., 2017). The role of PRC1 in the nervous system development is not well studied.

In this work, we show that *rnf2* loss of function is sufficient to perturb enteric neural crest migration and differentiation along the developing gut in zebrafish embryos. As a result, fewer enteric neurons were born in the ENS, leading to colonic aganglionosis. This clearly shows that the *rnf2* gene plays an important role in ENS development and highlights it as a novel candidate gene in Hirschsprung’s disease. We found that *rnf2* loss of function decreases the expression of *phox2b*, *ret*, *hand2*, and *crestin*. Previous reports have shown that in *Phox2b* knockout mice and zebrafish, ENS precursor cells can reach the foregut, but cannot continue to migrate or differentiate, and the expression of enteric neural precursor cell markers is abnormal (Fitze et al., 2010; Kwon et al., 2011). *Phox2b* is crucial to the formation of all autonomic ganglia, including enteric ganglia, and is suggested to promote the proliferation and survival of ENPCs. Consistently, its depletion leads to complete enteric aganglionosis (Alexandre et al., 1999). Interestingly, *phox2b* was ectopically expressed in the brain regions of *rnf2* mutant embryos. In zebrafish and mice, knockout of the *ret* gene leads to abnormal development of ENS (Carrasquillo et al., 2002; Carniti et al., 2006). The expression of early neural crest markers such as *foxd3*, *nestin*, *sox9b*, and *sox1b*, was altered in the absence of *rnf2*. The results of *in situ* hybridization showed that *rnf2* mutation disrupts the migration of ENPCs to the gut; the immunofluorescence assay showed that *rnf2* mutation results in the decrease of enteric neurons. These results suggest that *rnf2* loss of function leads to deficits in the specification of NCs, and affects the differentiation of subsequent enteric neurons by regulating the migration of ENPCs.

Mutations in PRC1 members such as *PHC1* and *PCGF2* lead to human neurodevelopmental disorders (Awad et al., 2013; Fitzgerald et al., 2015). Patients with *RING1* mutation have neurological psychosis, developmental abnormalities and cognitive impairment (Pierce et al., 2018). A recent study has shown that RING1 p.R95Q, which alters a conserved arginine residue in the catalytic RING domain, results in syndromic neurodevelopmental disabilities of a 13-year-old girl (Pierce et al., 2018). These observations suggest that RING1 also plays a critical role in CNS development beyond ENS. In order to study the function of *rnf2* in zebrafish CNS development, we compared the neuronal differentiation in control and mutant embryos. The results of immunofluorescence further showed that 5-HT^+^ neurons were mis-localized and displayed a dispersed manner. In 72 hpf *rnf2*^–/–^ embryos, Mbp-GFP signals in these regions are reduced and mis-localized. This result shows that knockout of *rnf2* gene also disrupts the development of myelinated glial cells (von Jonquieres et al., 2013). We also showed that formation of adrenergic and noradrenergic neurons was abnormal in *rnf2*^–/–^ embryos. This observation indicated that *rnf2* is required for proper differentiation of neurons in the CNS (Kousa et al., 2019; Song et al., 2020). Taken together, we concluded that *rnf2* is important for the proper migration and differentiation of neural precursor cells, and its absence leads to abnormal development of ENS and CNS in zebrafish.

## Data availability statement

The original contributions presented in this study are included in the article/Supplementary material, further inquiries can be directed to the corresponding authors.

## Author contributions

YS: conceptualization and funding acquisition. GF: formal analysis, investigation, resources, and visualization. GF and YS: manuscript. Both authors have read and agreed to the published version of the manuscript.

## Footnotes

1
https://asia.ensembl.org/Danio_rerio/Gene/Sequence?db=core;g=ENSDARG00000091029;r=14:17064126-17072736
