# Supplementary material for: The Polycomb group gene rnf2 is essential for central and enteric neural system development in zebrafish
Source: Front Neurosci. 2022 Sep 1;16:960149. doi: 10.3389/fnins.2022.960149 (PMC9475114; doi:10.3389/fnins.2022.960149)
Supplement: Supplementary file 1 [file Table_1.docx]

**The Polycomb group gene** ***rnf2* is essential for** **central and enteric neural system development in zebrafish**

**Table S1 Primers used to amplify DNA template for Probe making**

|  | | Primers (5′-3′) |
| --- | --- | --- |
| *hand2* | F | TCGCTGTCATGAAGAACCCC |
|  | R | CGCCTTTCTTCTTTGGCGTC |
| *ret* | F | GTTTGACCGCAGTGAGGGTA |
|  | R | GCCTTCTGTGAGACCGTGAA |
| *nestin* | F | TACTTGGGCCGTGTGAAACT |
|  | R | GACTCTCCTCCAGACGTCCA |
| *sox1b* | F | GCGGCAGCAAAGTTAACCAA |
|  | R | CGAGGCGCTCATGTAACTCT |
| *sox9b* | F | CAGCCTGGATGCACAAACC |
|  | R | GAGAGTATGGATGTGAGCGGG |
| *neurod1* | F | CGAGCAGAGCCAGGAGAT |
|  | R | AGGGTGGTGTCAAAGAACG |
| *tfap2a* | F | AGCTCGAGCCTTGTATGCAC |
|  | R | GCTCTCCTGACTTTGTCGCT |
| *egr2b* | F | CCCTCTTGCCGATAGCATCT |
|  | R | GTTGGAAAAAGCCGGCGTAG |
| *phox2a* | F | CCGGACATCTACACGAGAGAG |
|  | R | GGGAATTTGATGACCACGCTG |
| *cxcr4a* | F | AGACAAGTACCGTCTGCACC |
|  | R | CGGGTTGAGACAGCAGTGAA |
| *prdm1a* | F | TTTTAAGCACCCGGCAGACT |
|  | R | GCTTGCTGCTTGGCTTCAAT |
| *vgll2a* | F | CCGGGCCACCCATAAAAGAG |
|  | R | CGTAACGGGGGTCAAAATGC |
| *egfp* | F | ATGGTGAGCAAGGGCGAGGAGC |
|  | R | ACGCTGCCGTCCTCGATGTTGT |
